# Loot

**Published:** 1872-03

**Authors:** 


### Loot.

***Cerebro-Spinal Meningitis.***

The rumor that epidemic cerebro-spinal meningitis has appeared in some portions of this State, and the announcement that some cases have been reported to the Board of Health, induces me to write a few lines, with the hope of calling the attention of medical practitioners, who may have the opportunity of seeing this disease, to some details of treatment, and especially to the use of the ordeal bean of calabar, which I believe has been of service to me in the management of a recent limited epidemic.

Since the beginning of November last I have treated forty cases of cerebro-spinal meningitis. Twenty-nine were well-marked typical cases. The ages of my patients ranged from 3 to 16 years. Four died, and one is not yet out of danger. As this epidemic occurred under peculiar circumstances, and presented many features which may be of interest to the profession, I propose to publish soon a full report.

The therapeutics of this disease are notoriously unsatisfactory. The mortality of reported epidemics ranges from 30 to 75 per cent. The death-rate of my cases is only 10 per cent. But their number is too small to allow any legitimate conclusion to be drawn from such limited statistics as to the value of any particular treatment. I anticipate my report, therefore, by this preliminary statement, that others may not miss an opportunity of testing the value of the remedies I have employed.

Waiving for the present all discussion concerning the nosology, etiology, pathology and morbid anatomy of cerebro-spinal meningitis, I may be permitted to state that the most salient and central feature of this disease seemed to me to be an enormously increased reflex irritability of the nervous system, with an attendant paralysis of some branches of the sympatheticus. In studying my first case it occurred to me that calabar bean, which ophthalmologists use in eye-diseases dependent upon an irregular or interrupted innervation of the dioptric apparatus, and which lately has acquired considerable reputation in the treatment of various forms of tetanus, might be of use in this disease, the symptomatology of which is so largely made up by paretic and tetanoid phenomena.

At first I used the tincture of calabar bean, but afterwards I succeeded in procuring the extract. The extract was manufactured by Hazard and Caswell, 2½ drachms of which are equal to 2 ounces of the bean. I had one drachm rubbed up in glycerine and gave 2–5 drops at a dose.

I administered this drug in every case, as soon as tetanic contractions or apparent paralysis of the sphincter iridis appeared; except in two fatal cases, one of which terminated very rapidly, the other occurred before I had obtained the extract and after the tincture was exhausted.

In the great majority of cases the drug seemed to exert a favorable influence upon the symptoms which it was intended to control. In a few of the milder cases, where there was rather a contracted pupil, I administered atropia in pretty full doses, without, however, influencing the general course of the disease.

In the onset of the attack the symptoms are generally so violent, cephalalgia and rachialgia so severe and distressing, the skin and mucous membranes so congested, that depletion seems the one thing indicated, and yet I have resisted every temptation to abstract blood. I have neither bled, nor leeched, nor cupped, nor blistered. Ice in bladders to head and neck mitigated the pain and proved grateful to the patient.

Cathartics I used very moderately, not intending to produce any revulsive effect upon the bowels.

I had no reason to be dissatisfied with this treatment, when afterward, in some of the protracted cases, a prostration set in as extreme as the precedent violence, and a degree of emaciation followed which rivaled the marasmus of the everlasting gastro-intestinal catarrh of starveling foundlings.

In the first days, or whenever, in the course of the disease, there was great restlessness, or violent jactation, I administered moderate doses of chloral hydrate, 10–15 grains, repeated as required. Morphine I gave only in one case where ice seemed unpleasant and headache was worse.

Although there is a certain rhythm and periodicity in the progress of this disease, I have seen no reason for giving quinine until after convalescence was established, and then, in combination with iron, it doubtless proves beneficial in re-establishing the vigor of the system.

In most of the cases periods of coma follow, or alternate with spasms and jactations. In such cases I have found the iodide of potassium of service. But this drug must be administered with a bold and liberal hand. I have given from two scruples to a drachm in 24 hours; generally alternating it with the extract of calabar bean, so that my patients received every two hours either a dose of calabar (2–5 drops) or a dose of pot. iod. gr. 6–10.

Cerebro-spinal meningitis is not a self-limited disease; it is protean in its manifestations; probably more frightful in its phenomena than severe in its anatomical lesions,—at least in a majority of cases,—and offers, therefore, a hopeful field to therapeutists, although few victories have as yet been won. To induce physicians to make new trials I have ventured to state briefly the method of treatment I have pursued; so far as its results go, I believe they are more favorable than any that have been obtained.

In general hygiene little could be done in the present state of our knowledge as to the cause of the disease. I have used carbolic acid as a disinfectant, and internally to some extent.

In concluding these lines I wish to express my gratitude to Professor Jacobi, who had the extreme kindness to visit my patients with me, and to whose profound knowledge of the diseases of childhood and skillful application of modern therapeutics I am indebted for much valuable advice.—C. F. RODENSTEIN, M.D.—*Correspondence Med. Record, N. Y.*

***Report on Vaccination.***

The daily observations and experience of physicians have, for seventy years, uniformly proved that in their personal exposure to the sick of small-pox, and by the severest testing of the attendants of the sick in hospital, sick-room, and ambulance, thorough vaccination continues to give full protection against the contagion, for they are freely exposed to its presence, and even to its touch, without harm. Thorough vaccination is thorough protection against small-pox, and is as effective now as in the time of Jenner. It is generally admitted, and clearly proved, that thorough vaccination protects against small-pox without danger to life or diffusing any infection. Scarred and disfigured faces are rarely met with at the present day, except in persons who, having neglected to be vaccinated, have taken small-pox in the natural way. In proof of this protective influence of vaccination, and the liability to infection without it, we may mention that in a severe epidemic in Norwich, England, of 306 persons in several families, there were among 91 vaccinated persons, two cases of mild varioloid, and no deaths; and of 215 unvaccinated persons, 200 took the disease, and 46 of them died.

Revaccination is recommended, not only as a measure of renewed security against small-pox, but as a test of the completeness of the first vaccination. It is necessary to test the completeness of protection and to exhaust the receptivity of small-pox, and of vaccination itself. He who is insusceptible to one or more revaccinations with fresh virus, may generally deem himself as secure as vaccination, or even an attack of small-pox itself, can make him.

Small-pox, it is true, occurs occasionally in persons who have been once vaccinated; but its occurrence is very rare in those recently vaccinated, or revaccinated once or oftener, or in those in whom the first vaccination was thoroughly good, as judged by the Jennerian rules for vaccination. In these instances the disease manifests itself oftenest in mitigated form, known as varioloid, and the protection afforded by vaccination is found to be equal to that which is conferred by a previous attack of small-pox. It is believed that a revaccination will destroy any susceptibility to small-pox infection which may remain from incompleteness or imperfection of the primary vaccination. In the army of Bavaria revaccination has been compulsory since 1843, and from that date until 1857—a period of 14 years—not even a single case of unmodified small-pox occurred, nor a single death from the disease. The vaccine virus now in use in New York City is justly regarded by the most experienced physicians as trustworthy and effective, and affords all the necessary protection. Vaccine virus is not a carrier of other diseases. To a neglect of reasonable precautions, and to carelessness in the performance of the first vaccination, are due the unmerited discredit into which this greatest boon of a merciful Creator has occasionally fallen, and the fatal objection of certain classes to its employment. With due precautions, no other disease than that of vaccinia will be communicated. The distinguished English vaccinators, Dr. Marson, in 40,000 vaccinations; Dr. Leese, in as many more; Sir Wm. Jenner, in 13,000 sick children and adults in London, and Dr. West, of the Children's Hospital, in 26,000 children—all concur in saying that they have never seen any other diseases communicated with the vaccine, and that they disbelieve the popular reports that diseases are so communicated, nor is the child from whom the lymph is taken found to be suffering from the disease it is said to have imparted. Fresh lymph is always to be preferred when it can be obtained; but crusts may be used if such lymph is not to be had, and there is immediate necessity for vaccination. When practicable, it is desirable to vaccinate from arm to arm, with virus taken on quill-slips directly from a healthy individual to the arm of another.

Vaccination, by preventing small-pox, indirectly diminishes scrofulous diseases; and the death-rate of most constitutional diseases, to say nothing of small-pox itself, has materially decreased as the practice of vaccination has increased; and not only has mortality been diminished, but life has been lengthened and population augmented. The exact reverse is true of the former practice of inoculation of small-pox.

There is no age at which a person should or should not be vaccinated, when a necessity exists for immediate protection against small-pox; but it is best done in early childhood, for then the irritation is less, and the child unable to relieve itself by scratching the part, which, with the irritation of the clothes, often interrupts the progress of the vesicle, causes it to discharge prematurely, and become inflamed; whereby a vaccination, which might have run its course naturally, becomes altered, and an inflamed angry sore is produced, leaving a deformed and ill-looking scar and a diminished protection. Matter, too, taken from such a sore is worse than valueless. The age of three months is, on the whole, to be preferred. The most distinguished English authorities in vaccination, Simon, Marson, Seaton, and others, recommend, as preferable, and as ensuring greater ultimate protection, four or five separate good vesicles; and of these, in taking virus for the purpose of vaccinating others, one at least should remain untouched.

The utmost care should be taken, in the performance and management of a first vaccination, to make it as nearly perfect, regular, and exhaustive as possible; for upon this depends very much the after-susceptibility to small-pox, and it is very doubtful whether any amount of subsequent revaccination will fully compensate for the deficiency of the first.

The period for revaccination may be determined by circumstances; it never can be done too early or too promptly in persons exposed to small-pox, and it should be performed in every person soon after the age of puberty, however frequently it may be repeated at earlier or later periods of life. It may succeed at one time when it would not at another; indeed, it appears to be true that revaccination, as well as primary vaccination, actually takes more readily in some years than in others. "One thoroughly good vaccination to start with," says Seaton, "and one careful revaccination after puberty, are all that is necessary for protection as complete as any known proceeding can give against small-pox." After the twenty-fifth year of age the liability to small-pox in the well vaccinated is very small. Of the nearly 50,000 revaccinations in the Prussian army in the year 1833, only about one-third were perfectly successful; resembling, that is, the results of a primary vaccination so closely as scarcely to be distinguished from it; the remaining two-thirds being more or less modified, or failing entirely. In other trials on a large scale the success has not been as great. It should be remembered that secondary small-pox occurs on an average in about one per cent. of cases; and that while we urge revaccination both as a test of remaining receptivity to it and of further exhaustion of what may remain, it is well known that a single well-performed vaccination, perfect in all respects, with few exceptions, suffices to secure for life an individual subjected to it. The small-pox has lost none of its malignity and virulence, and while unvaccinated persons are allowed to accumulate, it is vain to hope for any exemption from epidemics of greater or less extent. In times of epidemic prevalence of small-pox, all should encourage vaccination. Vaccination and revaccination are positive duties. In order to give complete and assured protection against small-pox, every person not recently and thoroughly vaccinated should be at once revaccinated, and in subsequent life should repeat this duty as often as every five or six years, until in adult life the repeated revaccination ceases to have effect.—Dr. ROBERTS—*Med. Record.*

### *Hydrate of Chloral as a Rubefacient and Escharotic.*

A correspondent of the *Scientific American* strongly recommends the hydrate of chloral as a rubefacient and escharotic. He says: "When applied, the burning is precisely like that produced by a cataplasm of strong mustard; but, at the same time, a sedative action is perceived, which somewhat neutralizes the smarting, while it does not prevent an excessive irritation of the skin. It does not blister, but the part the chloral has been applied to becomes exceedingly inflamed, and more or less swollen, and, according to the length of time of application, shows a merely reddened skin, or a suppuration of several weeks' duration.

"I give you my mode of application: Take a piece of fresh adhesive plaster, of the size wanted, and crush fine, on its surface, with an ivory spatula, enough of the crystals of the chloral to powder the piece of adhesive plaster quite evenly; use the edge of the spatula to take off the chloral where it is more than a mere dust in thickness, but distribute evenly, leaving one-third of an inch margin for adhesion; heat the back of the plaster for an instant only, and apply. Leave it on about half an hour as a rubefacient, six hours as an irritant. To produce suppuration, put the chloral on the plaster in large quantities, and leave on from twenty-four to thirty-six hours; on its withdrawal, apply a stimulating salve, and afterwards heal with cerate. For an escharotic effect apply the chloral, thickly spread, and, after twelve hours, repeat the application, if necessary.

"It is surprisingly active as a suppurative, and for this reason it will not be prudent to apply it to any part of the surface of the abdominal cuticle, as, in one case under my inspection, having been left on too long, it occasioned a deep ulceration that was difficult to heal."

In this new mode of use, the chloral is truly invaluable, it is easily applied, cleanly, and perfectly reliable.—*Boston Journal of Chemistry.*

### *A Plan for Facilitating the Reduction of Strangulated Inguinal Hernia by Taxis.*

A correspondent of the *British Med. Journal* calls attention to a method recommended by Baron Sentin to be followed in this operation. The patient is to be placed on the back, with the hips well raised, and in case the hernial tumor is not reducible by the ordinary manipulation, the surgeon is to pass his index finger up into the ring, if possible to the outer side of the gut. After crowding the point of the finger past the column of the ring, strong and constant pressure is made, until the fibres of the ligament give way, or the opening is perceptibly enlarged, when it will be found that the intestine can be returned easily to the abdomen by the usual effort. The conditions which contraindicate a resort to this procedure, are:

1. The fact of the hernia being old and irreducible.
2. The presence of the constriction at the inner ring—the external ring being too small, and the canal too long, to admit the finger.
3. The existence of general symptoms of gangrene of the intestine.—*Med. Record.*

